# Glial acetate metabolism is increased following a 72-h fast in metabolically healthy men and correlates with susceptibility to hypoglycemia

**DOI:** 10.1007/s00592-018-1180-5

**Published:** 2018-06-22

**Authors:** David Harry McDougal, Moses Morakortoi Darpolor, Marina Andreyevna DuVall, Elizabeth Frost Sutton, Christopher David Morrison, Kishore Murali Gadde, Leanne Maree Redman, Owen Thomas Carmichael

**Affiliations:** 1 Pennington Biomedical Research Center, Baton Rouge, LA 70808 USA; 2 Present Address: Magee-Womens Research Institute, 204 Craft Ave, Pittsburgh, PA 15213 USA

**Keywords:** Humans, Fasting, Magnetic resonance spectroscopy, Hypoglycemia, Neuroglia, Glucose, Acetates

## Abstract

**Aims:**

Prior exposure to insulin-induced hypoglycemia was shown to increase glial acetate metabolism (GAM) during subsequent exposure to hypoglycemia in diabetic individuals. However, it remained unclear whether this effect was dependent on the disease state or the antecedent cause of hypoglycemia. We aimed to establish whether exposure to fasting-induced hypoglycemia was sufficient to produce alterations in GAM in non-diabetic individuals.

**Methods:**

GAM was measured via carbon-13 magnetic resonance spectroscopy during infusion of [1-^13^C] acetate before and after a 72-h fast in six metabolically healthy men. All participants were male, aged 18–40 years, with a Body Mass Index of 20.0–27.9 kg/m^2^, who consented to reside at Pennington Biomedical Research Center for 4 days. The main outcome measure was the percent enhancement of cerebral [1-^13^C] bicarbonate (the primary metabolic byproduct of glial oxidation of [1-^13^C] acetate). Continuous glucose monitoring was used to measure hypoglycemic episodes during the 72-h fast.

**Results:**

As expected, 72 h of fasting significantly reduced blood glucose levels and resulted in a high frequency of hypoglycemic episodes. Steady-state GAM increased from 53.5 ± 3.7 to 61.9 ± 1.7% following the 72-h fast (*p* = 0.005). This increase correlated with greater duration of hypoglycemia experienced during the fast (*r* = 0.967). In addition, subjects with greater GAM at baseline experienced a greater increase in the duration of hypoglycemia experienced during the 72-h fast (*r* = 0.979).

**Conclusions:**

GAM has potential as a biomarker for susceptibility to hypoglycemic episodes.

**Trail registration:**

Clinicaltrials.gov ID: NCT02690168.

**Electronic supplementary material:**

The online version of this article (10.1007/s00592-018-1180-5) contains supplementary material, which is available to authorized users.

## Introduction

Hypoglycemia-associated autonomic failure (HAAF) is a serious condition characterized by drastically reduced neuroendocrine responses to hypoglycemia, and the loss of physiological symptoms of hypoglycemia, or hypoglycemia unawareness [1]. The development of HAAF can lead to ever-worsening and often life-threatening episodes of severe hypoglycemia [2]. HAAF is a significant impediment to the maintenance of healthy plasma glucose levels in both type 1 and 2 diabetes [3, 4].

Glial cells are gaining acceptance for their regulatory functions in a variety of neurological conditions [5], including abnormal hypoglycemic counter-regulation [6–11]. Furthermore, neurospectroscopy studies have demonstrated alterations in glial metabolism in persons with diabetes (PWD) [12] and PWD with HAAF [13]. These studies assessed glial metabolism using carbon-13 magnetic resonance spectroscopy (^13^C MRS) combined with the infusion of ^13^C acetate (a monocarboxylate preferentially metabolized by glial cells) [14, 15]. Using this technique, Mason et al. [12] determined that glial acetate metabolism (GAM) during a hypoglycemic clamp was greater in PWDs than it was in controls. This was reflected by an increase in both the cerebral transport and acetate oxidation in diabetic subjects during hypoglycemia, yet these differences could not be directly attributed to the diabetic state per se. This is because the PWDs in this study displayed significantly reduced plasma levels of counter-regulatory hormones during the hypoglycemic clamp, indicating a high prevalence of HAAF in these subjects.

In a subsequent investigation by Gulanski et al. [13] GAM was assessed in PWDs with and without HAAF during a hypoglycemic clamp. GAM was greater in PWDs with HAAF than in those without HAAF and the non-diabetic control groups [13]. GAM was not different between PWDs without HAAF and controls. The authors suggest that in response to recurrent exposure to low glucose availability, individuals with HAAF may adapt by increasing their utilization of alternative fuels, such as acetate. Together, these results suggest that prior exposure to insulin-induced hypoglycemia in the diabetic disease state is associated with elevated GAM, but that diabetes itself is not sufficient to increase GAM.

Although consensus exists that HAAF is associated with abnormal GAM, it is unclear if hypoglycemia, independent of diabetes, can alter GAM. Furthermore, it is not understood if alterations in GAM are driven by exposure to hypoglycemia directly, as opposed to indirectly through treatment-induced hyperinsulinemia (the underlying antecedent of HAAF). The current study in metabolically healthy male subjects was, therefore, undertaken to understand these issues by using prolonged fasting as an alternative antecedent to hypoglycemia. A 72-h fast is known to produce moderate-to-severe hypoglycemia [16, 17] as well as hypoinsulinemia [16, 18, 19]. We hypothesized that in non-diabetic subjects exposed to a 72-h fast, hypoglycemia will induce increased GAM and any observed increase in GAM will be related to the magnitude of the hypoglycemia experienced during the fast.

## Materials and methods

### Subjects

An a priori power analysis (with a mean and standard deviation of 5 and 2.3, respectively) previously reported in a similar study [13] estimated that a sample size of 6 would provide adequate statistical power (0.85) for our primary outcome variable. Due to known gender differences in the counter-regulatory response to hypoglycemia [18, 20, 21], study enrollment was limited to males 18–40 years old with a Body Mass Index (BMI) between 20.0 and 27.9 kg/m^2^, who were willing to reside on the inpatient unit for 4 days. Subjects were excluded for prior diagnosis of type 1 or 2 diabetes mellitus, fasting glucose ≥ 110 mg/dL, hyperketonuria (≥ 15 mg/dL), contraindication to MRI, current use of prescription medications, history of or current eating disorder, history of obsessive compulsive disorder, contraindication to prolonged fasting, and consumption of ≥ 10 alcoholic drinks per week.

### Study design

The study was conducted over 6 months from 11/2016 to 04/2017. A graphical representation of the study design is presented in Figure S1. Recruited subjects were evaluated for eligibility at a single screening visit. Eligible subjects were then scheduled for a 4-day (~ 80 h) inpatient stay. Subjects arrived at the inpatient unit in the morning after a 10-h overnight fast (day 0) and weight, vital signs and body composition (Lunar iDXA whole-body scanner, General Electric; Milwaukee, WI, USA) were measured. GAM was then measured via ^13^C MRS, after which subjects were fed and began a ≈ 75-h fast when only water could be consumed ad libitum. Continuous glucose monitoring (CGM) was utilized for the duration of the 72-h fast and CGM glucose levels were recorded every 5 min. At 72 h, measurement of GAM was repeated.

### Clinical chemistry

Plasma or serum levels of blood glucose (BG), beta-hydroxybutyrate (BHB), free fatty acids (FFA), glucagon, insulin, epinephrine and norepinephrine levels were measured by the Pennington Biomedical Clinical Chemistry Core. See supplementary materials for further details of the specific methods used.

### GAM

Glial acetate metabolism was determined via MRS performed on a GE 3T Signa HDxt magnet (GE Healthcare, Milwaukee, WI, USA) with a single loop (8-cm diameter) radiofrequency (rf) coil (Doty Scientific Inc., Columbia, SC, USA). The 8-cm rf coil was tuned and matched to ^13^C resonance frequency at 3 T, and pulse sequence calibrations were implemented on a 2-mM sodium [1-^13^C] acetate phantom and a 1.76-mM sodium [1-^13^C]bicarbonate phantom. Each participant was positioned in a ^1^H-quadrature head coil with the ^13^C rf coil placed against the participant’s cranium. Scout images were acquired to localize and identify occipital lobe of the brain. Specifically, a high-resolution T1-weighted sequence was used to acquire 5-mm-thick slices in the transverse plane. Higher order shimming routine was used to improve the linewidths of spectral resonances. Baseline MRS data was acquired prior to the start of infusion with a free induction decay (FID) chemical shift imaging [22] sequence by implementing the following parameters: time-to-repetition (TR) of 4 s, a flip angle of 75°, a spectral width of 2.2 kHz, 1024 sampling points, number of excitation (NEX) of 8, and 296 total number of scans. At the start of [1-^13^C] acetate infusion, the total number of scans parameter was changed to 1808 to acquire a separate dynamic scan over a 2-h time course. For each participant, a 400-mM sodium [1-^13^C] acetate was infused at a rate of 6 mg/kg (body weight) for the first 5 min followed by 3 mg/kg (body weight) for 55 min. Venous blood samples were obtained at 10-min intervals during the ^13^C MRS procedures, starting 20 min prior to [1-^13^C] acetate infusion, and were used for plasma ^12^C and ^13^C acetate determination via gas chromatography–mass spectrometry [(GC–MS); (see supplementary material for details of GC–MS methods)] Plasma fraction enrichment of ^13^C acetate was determined by dividing the plasma concentration of ^13^C acetate by total plasma acetate concentration {[^13^C acetate]/([^13^C acetate] + [^12^C acetate])}.

All spectral data were post-processed in jMRUI version 5.2 [23] and quantitation of metabolite peaks was performed in the time domain with the AMARES (Advanced Method for Accurate, Robust, and Efficient Spectral fitting) algorithm [24]. FID data were apodized with 2 Hz, fast Fourier transformed, and manually phased (zero-order phase). Confirmed bicarbonate resonance peak data was transferred into Matlab (MathWorks, Natick, MA, USA), normalized with respect to the total sum and fitted to a mono-exponential equation as previously described by Bluml et al. [25] (see supplementary materials for details of equations).

### CGM

A G4 Platinum CGM system (Dexcom, San Diego, CA, USA) was used per manufacturer’s instructions to measure interstitial glucose. The sensor was calibrated every 12 h via capillary BG measurements by finger stick using a HemoCue B-glucose analyzer (HemoCue, Brea, CA, USA). After completing the fast, CGM data was exported to Microsoft Excel to determine the mean, maximum, and minimum BG, as well as percent time spent ≤ 70 mg/dL and frequency of hypoglycemic episodes. A hypoglycemic episode was defined as three or more consecutive CGM glucose readings ≤ 70 mg/dL (Level 1) or ≤ 54 mg/dL (Level 2) [26].

### Statistics

Results are reported as mean ± SE. All statistical analyses were performed using SAS Version 9.4 (SAS Institute, Cary, NC, USA) via a linear mixed effect model for repeated measures. The mixed effect model allowed all subjects to be included in the analyses irrespective of missing MRS data (see “GAM” for details) and models the correlation between the same subjects using a compound symmetric covariance matrix. Two sample *t* tests, based on estimates from the mixed model, were used to determine differences between day 0 and day 3 levels of plasma levels of metabolites and hormones, steady-state PE of ^13^C bicarbonate, and metabolic modeling parameters. Distributional assumptions of the residuals from these models were investigated using both visual inspection as well as Shapiro–Wilks tests. Pearson’s correlation was used to determine the association between time ≤ 70 and steady-state PE of bicarbonate on days 0 and 3. For all statistical analyses, *p* values of < 0.05 were considered statistically significant.

## Results

Baseline characteristics and demographics of the subjects are presented in Table 1. Subjects experienced a statistically significant weight loss (3.7 ± 0.4%) following the 72-h fast. All subjects had normoglycemia (85.1 ± 2.8 mg/dL) on day 0 (following a 12-h fast). Blood glucose levels were significantly reduced on day 3 (following the 72-h fast), exhibiting a 16% decrease relative to values obtained on day 0. Serum concentrations of FFA, glucagon, and epinephrine were significantly increased on day 3 (Table 2). Serum norepinephrine and acetate levels, however, were not changed (Table 2). There was no evidence of hyperinsulinemia on day 0 and insulin significantly decreased in response to the 72-h fast with concentrations below the level of detection (2.0 µU/mL) for 5/6 subjects. Similarly, there was no evidence of ketosis on day 0; however, the 72-h fast significantly increased BHB measured in serum and urine (Table 2).

**Table 1:** Subjects demographics and characteristics

	Subjects
Sex	
Male, *n* (%)	6 (100)
Race	
Black or African–American *n*, (%)	3 (50)
White *n*, (%)	3 (50)
Age	28.7 ± 1.0
Weight (kg)	76.1 ± 5.6
BMI (at screening)	23.5 ± 1.1
Fasting glucose (mg/dL)	84.8 ± 3.7
Body fat by iDXA, %	17.6 ± 3.0
Weight loss after the 72-h fast (%)	3.7 ± 0.4

Data are *n* (%) or mean ± SE; BMI was calculated based on screening anthropometrics; weight and body fat reflect day 0 measurements

*SE* standard error, *BMI* Body Mass Index

**Table 2:** Levels of metabolites and hormones on day 0 and day 3, following a 12-h fast and 72-h fast, respectively

	Day 0 12-h fast	Day 3 72-h fast	*p* value
Glucose, mg/dL	85.1 ± 2.8	71 ± 2.0*	0.005
Free fatty acid, mmol/L	0.6 ± 0.1	1.1 ± 0.2*	0.015
Serum β-hydroxybutyrate, mmol/L	0.1 ± 0.0	2.3 ± 0.5*	0.008
Urine β-hydroxybutyrate, mmol/L	–	20 ± 13	
Glucagon, pg/mL	56 ± 5.2	88 ± 7.0*	0.012
Insulin, µU/mL	3.1 ± 1.0	–	
Epinephrine, ng/mL	50 ± 6.7	72 ± 4.8*	0.041
Norepinephrine, ng/mL	380 ± 63	540 ± 110	0.108
Acetate (12-C), µmol/L	152 ± 6.1	167 ± 20	0.106

Data are mean ± SE. Data omitted when values were below minimal detection limits of the assay

*SE* standard error

*Significant difference between 12- and 72-h values as determined by paired *t* tests, *p* value < 0.05

**Fig. 1:**
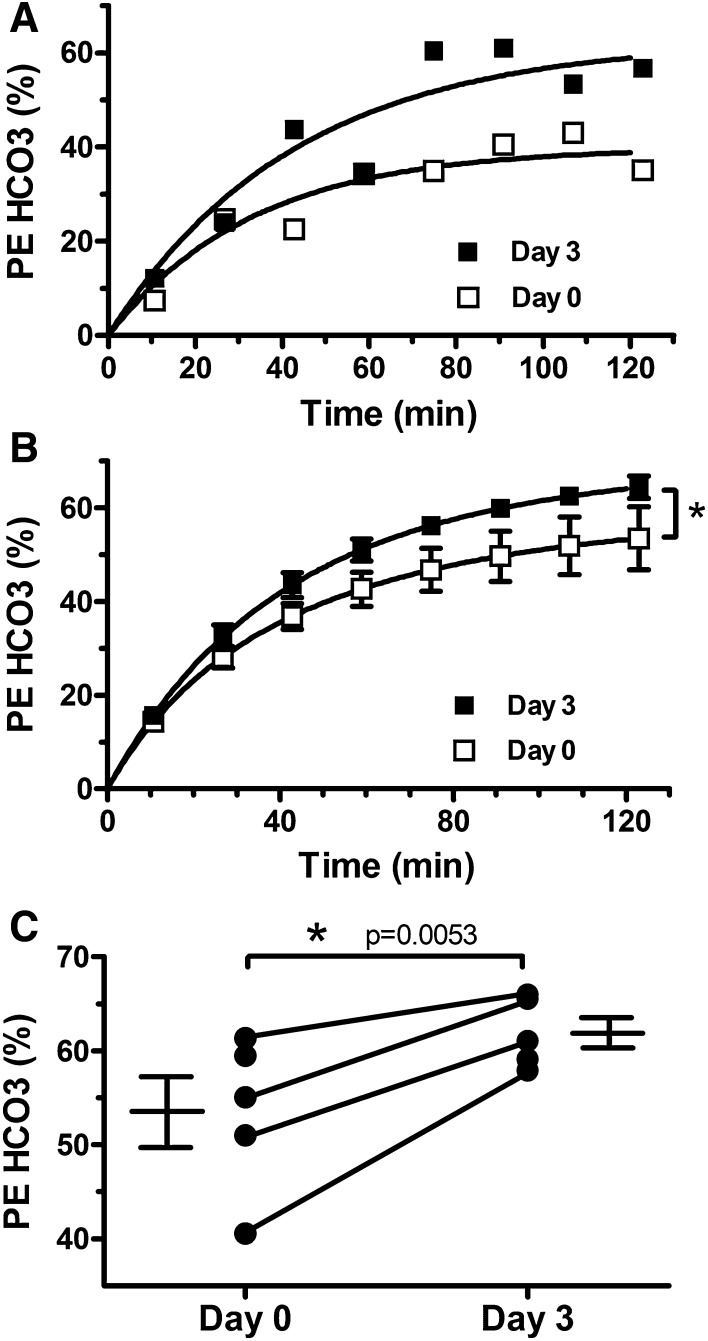
Glial acetate metabolism (GAM) is increased following a 72-h fast. GAM was measured via carbon 13 magnetic resonance spectroscopy during [1-^13^C] acetate infusion. **a** Time course of the percent enhancement (PE) of ^13^C bicarbonate (HCO_3_; the primary metabolite of [1-^13^C] acetate oxidation) following the start of infusion in a representative subject after 12 h of fasting (day 0) and 72 h of fasting (day 3). The solid line represents the best fit of a mono-exponential function modeling GAM. **b** Comparison of the average best fit across subjects via a linear mixed effects model demonstrates a clear increase from day 0 to 3 (*p* < 0.0001). **c** Average steady-state PE of HCO_3_ was increased on day 3 relative to day 0 (*p* = 0.0053). Furthermore, the PE of HCO3 was increased on day 3 relative to day 0 in all subjects who completed both scans (symbols with connecting lines)

[1-^13^C] acetate infusion produced a rapid increase in the plasma fractional enrichment of [1-^13^C] acetate within 10 min. Mean plasma fractional enrichment of [1-^13^C] acetate did not differ between day 0 and day 3 (70.32 ± 0.02 and 69.26 ± 0.02%, respectively; *p* = 0.61), nor did plasma levels of [1-^13^C] acetate (data not shown). Prior to the infusion of [1-^13^C] acetate, no spectral peaks associated with acetate metabolism were detected (Figure S2, panel A). With the onset of the [1-^13^C] acetate infusion, a rapid increase in the cerebral PE of ^13^C bicarbonate was observed (Fig. 1a). This enhancement reflects the glial oxidation of our infusate via the TCA cycle. Additional peaks associated with [1-^13^C] acetate oxidation by glial cells, including [1-^13^C] glutamine, [5-^13^C] glutamine, [1-^13^C] glutamate, and [5-^13^C] glutamate, were also observed in our spectra (Figures S2B and S2C). Total PE of ^13^C bicarbonate approached steady-state within 60–70 min (Fig. 1a) and persisted in our spectra following the offset of the [1-^13^C] acetate infusion (Figures S2B and S2C). Figure 1b displays the average curve-fit for all days 0 and 3 scans, respectively, at approximately 15-min intervals. A clear increase in the PE of ^13^C bicarbonate is shown in the average day 3 curve-fit relative to day 0, with a statistically significant increase in PE of bicarbonate on day 3 versus day 0 curve fits (*p* < 0.0001). The curve-fitting procedure also yielded the cerebral metabolic rate constants CMR_ACE_, V_TCA_, V_ACE_, and CO_2_ from Eq. (2) as described in supplimentary materials. There were no significant differences in the average values of these rate constants for day 0 versus day 3 (Table S1). Mean steady-state PE of ^13^C bicarbonate measured from 64 to 96 min following the initiation of the [1-^13^C] acetate infusion also increased on day 3 relative to day 0 (61.9 ± 1.7 versus 53.5 ± 3.7; *p* = 0.005). The day 0 values of PE of bicarbonate displayed increased inter-subject variability relative to day 3 values. Day 0 values ranged from 40.6 to 61.4%, while day 3 values ranged from 57.9 to 66.0% (Fig. 1c). Furthermore, in the 4 subjects with complete data on day 0 and day 3, PE of ^13^C bicarbonate was increased on day 3 relative to day 0 (filled circles with connecting lines in Fig. 1c). Altogether, these data reflect an increase in the rate of glial metabolism of [1-^13^C] acetate on day 3 relative to day 0.

**Fig. 2:**
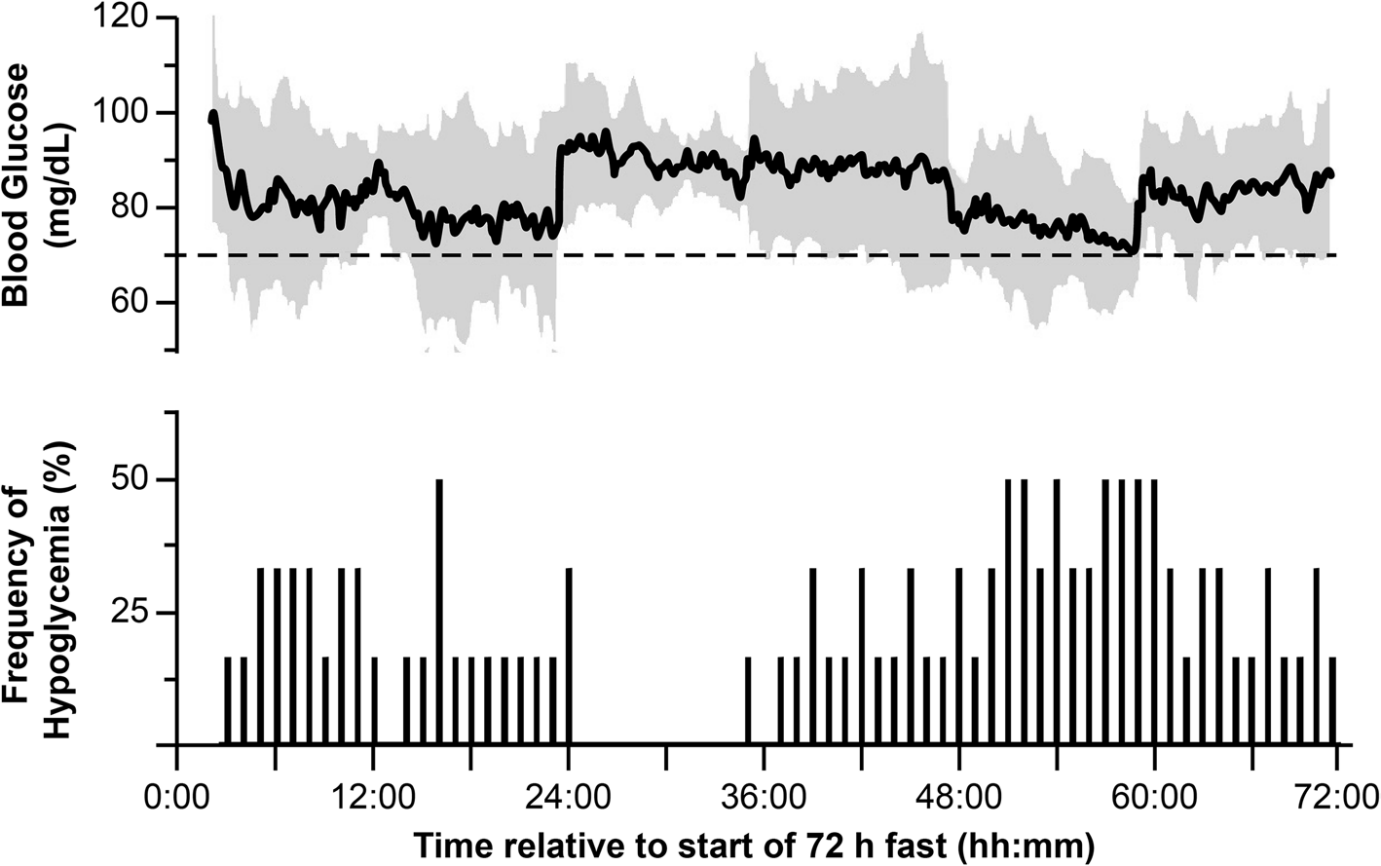
Continuous glucose monitoring (CGM) demonstrates that 72 h of fasting leads to frequent bouts of hypoglycemia. CGM was used to monitor blood glucose levels at 5-min intervals throughout a 72-h fast. The mean blood glucose for all six subjects is plotted in the top panel. The gray shading represents the 95% confidence limit and the dotted line represents the clinical threshold for Level 1 hypoglycemia, 70 mg/dL. The lower panel represents the percent of subjects which experienced a hypoglycemic episode during each hour of the 72-h fast. A subject was determined to have had a hypoglycemic episode during a given hour of the fast if a CGM reading of ≤ 70 mg/dL was maintained for three consecutive CGM readings (15 min)

**Fig. 3:**
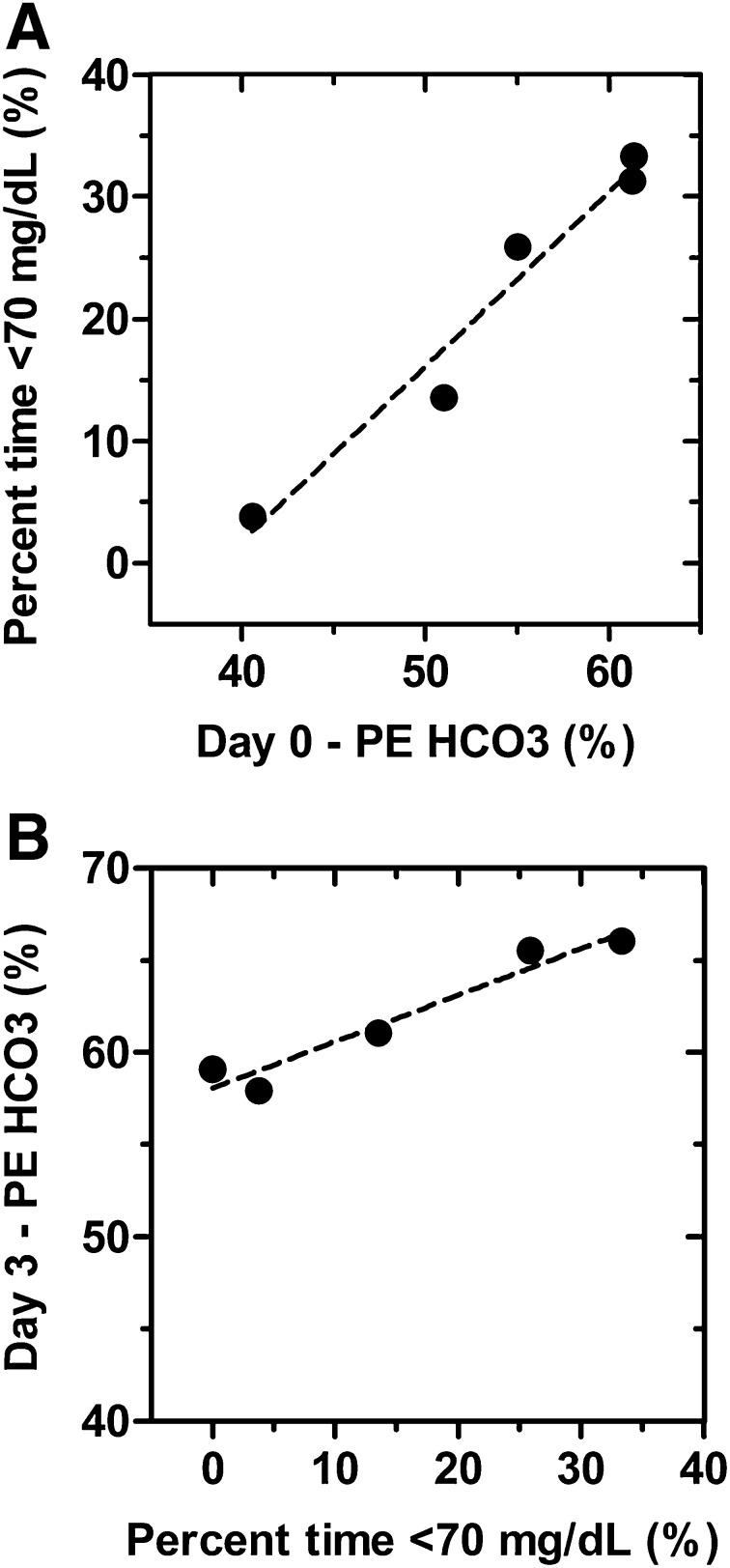
Individual differences in glial acetate metabolism (GAM) are related to the duration of hypoglycemia experienced during a 72-h fast. GAM, as measured by the percent enhancement of ^13^C bicarbonate (PE of HCO_3_) following an acetate infusion, is both predictive of and responsive to subsequent fasting-induced hypoglycemia. **a** Relationship between glia acetate metabolism measured just prior to 72 h of fasting (day 0) and the subsequent exposure to hypoglycemia during the fast (percent time ≤ 70 mg/dL). A correlation analysis between these two variables demonstrated a robust positive relationship between GAM and the duration of hypoglycemia experienced during fasting (Pearson’s *r*^2^ = 0.96, *p* = 0.004). **b** GAM measured following a 72-h fast (day 3) increase in a dose-dependent manner based on the magnitude of exposure to hypoglycemia during the fast (Pearson’s *r*^2^ = 0.94, *p* = 0.007)

CGM glucose values were not significantly different from BG values measured at identical time points (*p* = 0.1835; data not shown). CGM glucose levels dropped well below 80 mg/dL during the first 24 h of the fast. On day 2, glucose levels rebounded to approximately 90 mg/dL before falling again toward hypoglycemic levels (< 70 mg/dL) during the last 24 h (Fig. 2, top panel). The subjects’ mean CGM glucose level during the 72-h fast was well within the normal range (70–150 mg/dL). However, the mean minimum CGM reading was below 70 mg/dL (Table 3), and the subjects experienced considerable time below 70 mg/dL (Fig. 2). The number of subjects who experienced at least one episode of Level 1 hypoglycemia was five (83.3%), four (60%), and five (83.3%) during the first, second, and third days of fasting, respectively. The number of subjects who experienced at least one episode of Level 2 hypoglycemia was two (33.3%), zero (0%), and one (16.7%) during the first, second, and third days of fasting, respectively. The percent time spent in the hypoglycemic state (< 70 mg/dL) was positively associated with the PE of bicarbonate measured on day 0 (Pearson’s *r*^2^ = 0.96, *p* = 0.004; Fig. 3a). Furthermore, an increased exposure to hypoglycemia was positively associated with increased GAM at the end of the 72-h fast (Pearson’s *r*^2^ = 0.94, *p* = 0.007; Fig. 3b).

**Table 3:** Continuous glucose monitoring parameters during the 72-h fast

Mean blood glucose, mg/dL	84 ± 4.3
Maximum, mg/dL	118 ± 7.5
Minimum, mg/dL	51 ± 5.7
Time at < 70 mg/dL, %	18 ± 5.8
Time at < 54 mg/dL, %	3.5 ± 1.8

Data are mean ± SE or %

*SE* standard error

## Discussion

Alterations in GAM are associated with a variety of clinical conditions in humans [27] including abnormal hypoglycemic counter-regulation [12, 13]. In this study, we used ^13^C MRS in non-diabetic subjects to assess whether GAM increased following a 72-h fast (a natural initiator of hypoglycemia). The major findings of this study were: (1) a 72-h fast in metabolically healthy men increases GAM, (2) increased exposure to hypoglycemia during the fast is associated with larger increases in GAM, and (3) increased GAM measured prior to the 72-h fast is correlated with increased susceptibility to hypoglycemia during the fast. The first two findings support our original hypothesis, while the third finding was unexpected and suggests that ^13^C MRS is a promising marker for hypoglycemia risk.

Hypoglycemia is a major complication of type 1 and 2 diabetes. Recurrent exposure to insulin-induced hypoglycemia can lead to the development of HAAF (a life-threatening metabolic disorder associated with blunted hypoglycemic counter-regulation). Previous studies demonstrated that HAAF is associated with increased GAM in PWDs during acute hypoglycemia [12, 13]. Our results advance these finding by demonstrating that exposure to hypoglycemia alone (independent of diabetes or exposure to hyperinsulinemia) is sufficient to increase glial metabolism. Furthermore, using continuous glucose monitoring to document the magnitude of hypoglycemic exposure, we demonstrated for the first time that exposure to hypoglycemia leads to a dose-dependent increase in GAM.

Our observation that GAM may determine susceptibility to fasting-induced hypoglycemia is also noteworthy in light of previous work. Gulanski et al. [13] showed that during a hypoglycemic clamp, GAM in both PWDs and non-diabetic subjects was inversely related to their neuroendocrine responses to the clamp. Thus, subjects with increased GAM displayed reduced hypoglycemic counter-regulation, thereby demonstrating increased susceptibility to hypoglycemia. In addition, our finding regarding to the metabolic rate constants associated cerebral acetate metabolism (Table S1) are consistent with those of Gulanski et al. [13], with the exception that Gulanski et al. [13] did observe a 50% increase in CMR_ACE_. Altogether, our findings along with those of Gulanski et al. [13] suggest that increased GAM is a predictor of increased susceptibility to hypoglycemia independent of disease state, acute glycemic condition (hypoglycemic versus euglycemic), and the cause of the hypoglycemia (fasting or hyperinsulinemia). These data suggest that further investigations into the relationship between GAM and HAAF may provide key mechanistic and diagnostic insights which could lead to better treatment outcomes for PWD.

The clinical management of HAAF is severely hampered by the lack of non-invasive, objective biomarkers useful for definitive diagnosis and for ascertaining the severity of HAAF in PWD. Although our study was not performed in PWDs, our results are applicable, since the physiological response to fasting-induced hypoglycemia is identical to that of insulin-induced hypoglycemia [28], and exposure to fasting-induced hypoglycemia produces HAAF-like symptoms in humans [29, 30]. Our observation that GAM during euglycemia may predict subsequent susceptibility to hypoglycemia raises the possibility for targeted assessment of GAM to assess hypoglycemic risk in PWD.

Several limitations of our study are worth noting. Our study was adequately powered to detect our primary endpoint, change in PE of bicarbonate. Yet, the robustness of correlations between the PE of bicarbonate and hypoglycemia, as well as our CGM dataset, would be strengthened by an increased sample size. Although CGM is commonly used to clinically monitor hypoglycemia, CGM glucose levels are not as accurate as blood glucose measurements. This limitation was offset somewhat by our choice of the Dexcom G4 device, which has been shown to be significantly more accurate than comparable devices [31]. In addition, our use of [1-^13^C] acetate instead [2-^13^C] acetate precluded determination of brain ^13^C acetate concentrations due to the close spectral proximity of 1-^13^C acetate and 5-^13^C glutamine, a downstream metabolite of 1-^13^C acetate oxidation (Figure S2B).

## Conclusion

Using a unique natural paradigm to induce hypoglycemia in healthy men, and a sophisticated ^13^C MRS protocol, our study significantly advances the scope and importance of the relationship between GAM and hypoglycemia. Increases in GAM are directly proportional to the extent of hypoglycemia exposure, in a dose-dependent manner, and higher levels of GAM under euglycemic conditions are associated with susceptibility to hypoglycemia. These two findings are very important clinically, i.e., GAM is possibly a novel biomarker of hypoglycemic risk and its measurement by ^13^C MRS has the potential to provide a much-needed diagnostic tool for the detection and treatment of HAAF.

## Electronic supplementary material

Below is the link to the electronic supplementary material.

Supplementary material 1 (PDF 549 KB)

Figure S1. Study design including screening and inpatient study visit, as well as all procedures and meals. Abbreviations: WGT (weight), MRS (magnetic resonance spectroscopy scan), AEs (adverse events), EOS (end of study) (TIF 626 KB)

Figure S2. Representative spectra collected from the occipital/parietal cortex using carbon-13 magnetic resonance spectroscopy coupled with a 60-minute intravenous infusion of 1-^13^C acetate. Spectra were collected 20 minute prior to, and 120 minute following the start of the infusion. Each spectrum displayed represents the average data from a ≈20 minute time block. Spectral peaks corresponding to 1-^13^C acetate (Ac1), as well as the following downstream metabolites of 1-^13^C acetate oxidation are annotated: Gln5 (5-^13^C glutamine), Glu5 (5-^13^C glutamate), Gln1 (1-^13^C glutamine), Glu1 (1-^13^C glutamate), and HCO3 (1-^13^C bicarbonate). No natural abundance peaks corresponding to either acetate or its metabolites were detected in baseline spectra collected prior to the initiation of acetate infusion (A). All these peaks were all readily apparent during the [1-^13^C] acetate infusion and continued to be present shortly after termination of the [1-^13^C] acetate infusion (B). Spectra collected 90 minutes following the initiation of the infusion often lacked peaks for less abundant metabolites of 1-^13^C acetate oxidation, such as Glu5. In contrast, the peak for the major metabolite of 1-^13^C acetate oxidation, HCO3, continued to be present for the entire 120 minutes following the commencement of the infusion (C) (TIF 2148 KB)

Supplementary material 4 (DOCX 13 KB)
