# Supplementary material for: Glial acetate metabolism is increased following a 72-h fast in metabolically healthy men and correlates with susceptibility to hypoglycemia
Source: Acta Diabetol. 2018 Jun 22;55(10):1029–36. doi: 10.1007/s00592-018-1180-5 (PMC6153507; doi:10.1007/s00592-018-1180-5)
Supplement: Supplementary file 1 — Supplementary material 1 (PDF 549 KB) [file 592_2018_1180_MOESM1_ESM.pdf]

Supporting Information:

Additional Methods-

Clinical chemistry

Blood glucose (BG), serum beta-hydroxybutyrate (BHB), and serum free fatty acid (FFA) levels were measured via a glucose electrode (Beckman Coulter, Brea, CA) and enzymatic assays, respectively (BHB, Sigma, St. Louis, MO; FFA, Wako diagnostics, Richmond, VA), using a Beckman Coulter DXC600 clinical analyzer (Beckman Coulter, Brea, CA). Plasma glucagon, serum insulin, and plasma epinephrine and norepinephrine levels were measured via radiometric assay (Millipore, Billerica, MA), immunoassay, and ELISAs (DLD Diagnostika, Hamburg, Germany), respectively. In the collection tubes, 500 kIU/ml of aprotinin was added immediately after collection (glucagon) while 10% sodium metabisulfite was added prior to collection (epinephrine and norepinephrine).

Mathematical modeling of MRS data

Confirmed bicarbonate resonance peak data was transferred into Matlab (MathWorks, Natick, MA), normalized with respect to the total sum (Equation 1) and fitted with a mono-exponential equation (Equation 2).

$$PE(\%) = \left[\frac{Amplitude}{\sum Amplitude/10} \right] \times 100$$ Equation 1,

$$PE(t) = CMR_{ace} \times \left(1 - e^{\left(-1 \times \left(\left(V_x + V_{tca}\right)/CO_2\right) \times t\right)}\right)$$ Equation 2.

where the peak at each moment in time (min, t) is expressed as a function of the percent enhancement (PE, as time series), cerebral metabolic rate of acetate (μmol/g/min, CMR_{ace}), tricarboxylic acid cycle rate (μmol/g/min, V_{tca}), acetate utilization rate (μmol/g/min, V_x), and the decarboxylated carbon (μmol/g, CO_2) in the TCA cycle. In the process of fitting the exponential equation, nonlinear-least-squares method was used with the added option of robust-bisquare and trust-region algorithm. Interested readers can find addition details of this mono-exponential model, represented by equations 1 and 2, in a previously published work by Bluml et al [1]. Also, Mason et al [2] presents detailed models for acetate utilization in the brain.

GC-MS methods

20 μL of internal standard (50 mM sodium acetate-D_3 in water) and 10 μL of a saturated aqueous solution of 5-sulfosalicylic acid were added to each 300 μL aliquot of plasma in a microcentrifuge tubes. The mixtures were vortexed and then centrifuged for 5 minutes at 13.2 rpm. The supernatants were transferred to screw-top vials (necessary, due to the pressurization caused by the derivatization) and the protein pellets were discarded.

Derivatization was performed by a method adapted from Zheng et. al.[3] 250 μL of a 3:2 n-propanol/pyridine solution was added to the supernatants, followed by 50 μL propyl chloroformate. The reaction was vortexed briefly and sonicated for 30 to 60 seconds. 300 μL hexanes were added to each sample, which were then vortexed and allowed to sit at room temperature until the biphasic contents had settled. The top layer was transferred to an autosampler vial and stored at 4° C until analysis.

GC-MS analyses were performed on a 7809A GC system (Agilent) with 5975C MSD (Agilent). A 1 µL volume of sample was injected (inlet parameters: heater = 250° C; pressure = 25.153 psi; septum purge flow = 3mL/min; mode = splitless; purge flow to split vent =15 mL/min at 0.75 min). The column was a J&W 123-1364 60 m x 320 µm x 1.8 µm (Agilent) heated according to the following gradient: hold at 70° C for 2 min, ramp to 110° C at 3° C/min, hold 0 min, ramp to 225° C at 40° C/min, hold for 1 min (flow = 3.7168 mL/min; equilibration time = 0.5 min). The MS quad was set to 150 ° C and the MS source was set to 230° C. The solvent delay was set at 7 minutes, and the detector was set to shut off at 8.5 minutes (MS acquisition in full scan with gain factor of 10; scan speed = normal).

^{12}C and ^{13}C acetate abundances were measured by integrating the extracted ion chromatograms for the most abundant fragments from the MS (43.1 m/z and 44.1 m/z, respectively) and concentrations were calculated using a simple area ratio with respect to the internal standard (46.2 m/z) as shown in the equation below.

$$[analyte] = \frac{peak\ area\ of\ analyte * [internal\ standard]}{peak\ area\ of\ internal\ standard}$$

Table S1. Metabolic modeling parameters from MRS data curve fits

	Day 0	Day 3	p-value
CMR_{ACE} (μmol/g/min)	59 ± 9.3	71 ± 6.7	0.477
V_{TCA} (μmol/g/min)	0.5 ± 0.1	0.4 ± 0.1	0.917
V_{ACE} (μmol/g/min)	0.2 ± 0.1	0.3 ± 0.1	0.573
CO_2 (μmol/g)	31 ± 11	28 ± 5.5	0.794

Data are mean ± SE

MRS (magnetic resonance spectroscopy scan), CMR_{ace} (cerebral metabolic rate of acetate), V_{tca} (tricarboxylic acid cycle rate), CO_2 (decarboxylated carbon), SE (standard error)

Figure S1

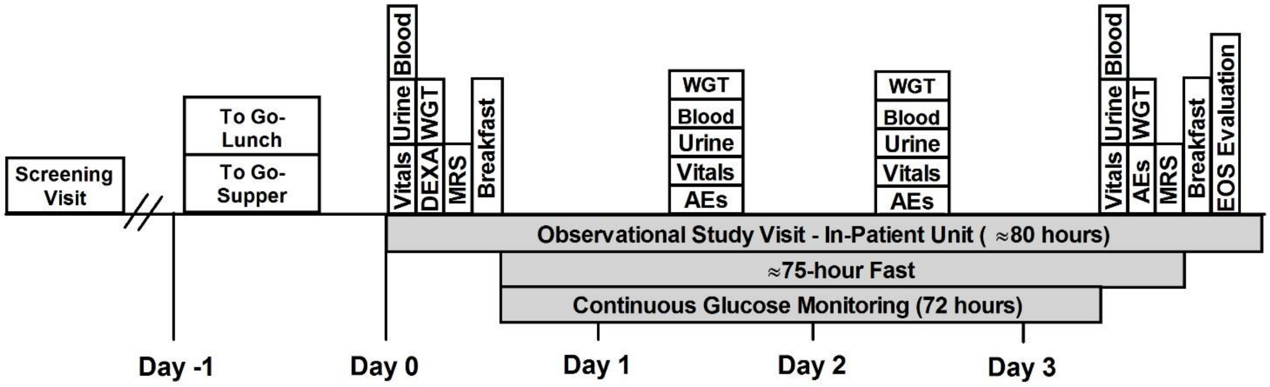

Figure S2

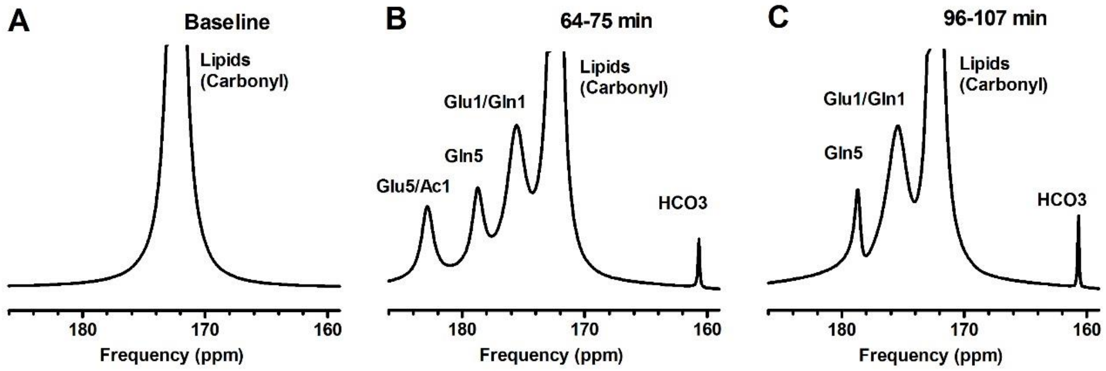

Figure legends

Figure S1. Study design including screening and inpatient study visit, as well as all procedures and meals

Abbreviations: WGT (weight), MRS (magnetic resonance spectroscopy scan), AEs (adverse events), EOS (end of study).

Figure S2. Representative spectra collected from the occipital/parietal cortex using carbon-13 magnetic resonance spectroscopy coupled with a 60-minute intravenous infusion of 1-^{13}C acetate. Spectra were collected 20 minute prior to, and 120 minute following the start of the infusion. Each spectrum displayed represents the average data from a ≈20 minute time block. Spectral peaks corresponding to 1-^{13}C acetate (Ac1), as well as the following downstream metabolites of 1-^{13}C acetate oxidation are annotated: Gln5 (5-^{13}C glutamine), Glu5 (5-^{13}C glutamate), Gln1 (1-^{13}C glutamine), Glu1 (1-^{13}C glutamate), and HCO3 (1-^{13}C bicarbonate). No natural abundance peaks corresponding to either acetate or its metabolites were detected in baseline spectra collected prior to the initiation of acetate infusion (A). All these peaks were all readily apparent during the [1-^{13}C] acetate infusion and continued to be present shortly after termination of the [1-^{13}C] acetate infusion (B). Spectra collected 90 minutes following the initiation of the infusion often lacked peaks for less abundant metabolites of 1-^{13}C acetate

oxidation, such as Glu5. In contrast, the peak for the major metabolite of 1-^{13}C acetate oxidation, HCO3, continued to be present for the entire 120 minutes following the commencement of the infusion (C).

References:

1. Bluml S, Moreno-Torres A, Shic F, Nguy CH, Ross BD. Tricarboxylic acid cycle of glia in the in vivo human brain. *NMR Biomed.* 2002; 15:1-5.

2. Mason GF, Petersen KF, Lebon V, Rothman DL, Shulman GI. Increased brain monocarboxylic acid transport and utilization in type 1 diabetes. *Diabetes.* 2006; 55:929-934.

3. Zheng X, Qiu Y, Zhong W, et al. A targeted metabolomic protocol for short-chain fatty acids and branched-chain amino acids. *Metabolomics : Official journal of the Metabolomic Society.* 2013; 9:818-827.
