# Supplementary figures and images for: Glial acetate metabolism is increased following a 72-h fast in metabolically healthy men and correlates with susceptibility to hypoglycemia
Source: Acta Diabetol. 2018 Jun 22;55(10):1029–36. doi: 10.1007/s00592-018-1180-5 (PMC6153507; doi:10.1007/s00592-018-1180-5)

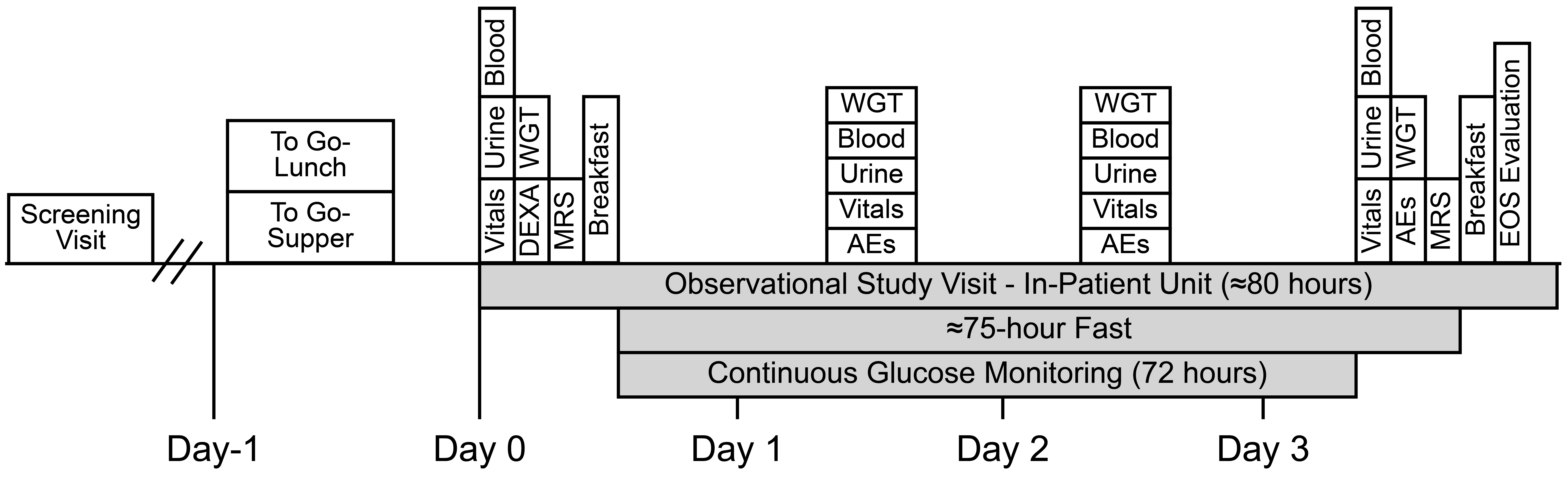

Supplement: Supplementary file 2 — Figure S1. Study design including screening and inpatient study visit, as well as all procedures and meals. Abbreviations: WGT (weight), MRS (magnetic resonance spectroscopy scan), AEs (adverse events), EOS (end of study) (TIF 626 KB) [file 592_2018_1180_MOESM2_ESM.tif]
