# Supplementary material for: Glial acetate metabolism is increased following a 72-h fast in metabolically healthy men and correlates with susceptibility to hypoglycemia
Source: Acta Diabetol. 2018 Jun 22;55(10):1029–36. doi: 10.1007/s00592-018-1180-5 (PMC6153507; doi:10.1007/s00592-018-1180-5)
Supplement: Supplementary file 4 — Supplementary material 4 (DOCX 13 KB) [file 592_2018_1180_MOESM4_ESM.docx]

| **Supplementary Table 1. Metabolic modeling parameters from MRS data curve fits** | | | |
| --- | --- | --- | --- |
|  | Day 0 | Day 3 | p-value |
| CMR_ACE_ | 59 ± 9.3 | 71 ± 6.7 | 0.477 |
| V_TCA_ | 0.5 ± 0.1 | 0.4 ± 0.1 | 0.917 |
| V_ACE_ | 0.2 ± 0.1 | 0.3 ± 0.1 | 0.573 |
| CO_2_ | 31 ± 11 | 28 ± 5.5 | 0.794 |
| Data are mean + SE  MRS (magnetic resonance spectroscopy scan), CMR*_ace_* (cerebral metabolic rate of acetate), V*_tca_* (tricarboxylic acid cycle rate), CO_2 (_decarboxylated carbon), SE (standard error) | | | |
